# Combined Effects of Eicosapentaenoic Acid and Adipocyte Renin–Angiotensin System Inhibition on Breast Cancer Cell Inflammation and Migration

**DOI:** 10.3390/cancers12010220

**Published:** 2020-01-16

**Authors:** Fahmida Rasha, Chanaka Kahathuduwa, Latha Ramalingam, Arelys Hernandez, Hanna Moussa, Naima Moustaid-Moussa

**Affiliations:** 1 Department of Nutritional Sciences, Texas Tech University, 1301 Akron Ave, Lubbock, TX 79409, USA; 2 Obesity Research Institute, Texas Tech University, Lubbock, TX 79409, USA; 3 Department of Psychiatry, School of Medicine, Texas Tech University Health Science Center, Lubbock, TX 79430, USA; 4 Department of Mechanical Engineering, Texas Tech University, Lubbock, TX 79409, USA

**Keywords:** obesity, breast cancer, renin–angiotensin system, eicosapentaenoic acid, adipocyte inflammation

## Abstract

Obesity is a major risk factor for breast cancer (BC). Obesity-related metabolic alterations such as inflammation and overactivation of the adipose renin–angiotensin system (RAS) may contribute to the progression of BC. Clinically used antihypertensive drugs such as angiotensin-converting enzyme inhibitors (ACE-I) and dietary bioactive components such as eicosapentaenoic acid (EPA) are known for their anti-inflammatory and adipose RAS blocking properties. However, whether EPA enhances the protective effects of ACE-I in lessening adipocyte inflammation on BC cells has not been studied. We hypothesized that combined EPA and ACE-I would attenuate BC cell inflammation and migration possibly via adipose RAS inhibition. To test our hypothesis, we examined the (i) direct effects of an ACE-I (captopril (CAP)) or EPA, individually and combined, on MCF-7 and MDA-MB-231 human BC cells, and the (ii) effects of conditioned medium (CM) from human adipocytes pretreated with the abovementioned agents on BC cells. We demonstrated that CM from adipocytes pretreated with EPA with or without captopril (but not direct treatments of BC cells) significantly reduced proinflammatory cytokines expression in both BC cell lines. Additionally, cell migration was reduced in MDA-MB-231 cells in response to both direct and CM-mediated CAP and/or EPA treatments. In summary, our study provides a significant insight into added benefits of combining anti-inflammatory EPA and antihypertensive ACE-I to attenuate the effects of adipocytes on breast cancer cell migration and inflammation.

## 1. Introduction

Breast cancer (BC) is the most common type of cancer among U.S. women and has a lifetime risk of more than 12% [1]. Obesity is one of the major modifiable risk factors for BC, especially in postmenopausal women, and is associated with poor cancer outcomes and survival in patients with BC [2,3]. Moreover, as inflammation is an important underlying basis for both diseases, it is critical to understand its involvement in mechanism(s) of obesity-related BC [4]. In obesity, adipose tissue expands and becomes hypertrophic and hypoxic, with invasion of proinflammatory M1-type macrophages and exaggerated secretion of protumor adipocytokines [5]. Various proinflammatory adipocytokines such as interleukin (IL)-6, IL-8, and monocyte chemoattractant protein-1 (MCP-1) can promote the activation and transcription of many protumor signaling pathways such as nuclear factor kappa B (NF-κB) and signal transducer and activator of transcription 3 (STAT3), resulting in increased migratory and invasive capacities in the adjacent cancer cells [6]. Additionally, cancer-associated adipocytes form a proinflammatory microenvironment via induced macrophage invasion and thus synergistically activate STAT3 and NF-κB pathways [7], which in turn leads to apoptosis inhibition and cancer cell proliferation [8]. Moreover, aberrant STAT3 signaling also upregulates angiogenic vascular endothelial growth factor (VEGF), leading to higher tumor angiogenesis [9]. Hence, obesity-associated proinflammation increases cancer cell progression via their interaction with adjacent adipocytes [6]. Furthermore, obese adipocytes also overexpress metabolic markers such as fatty acid synthase (FASN), which is associated with adipocyte hypertrophy and increased proinflammatory adipocytokine secretion in obesity [10,11]. FASN provides an alternative energy source to proliferating cancer cells in the form of free fatty acids helping their growth and proliferation [12]. This, in turn, facilitates the formation of a toxic tumor microenvironment (TME) in the breast, which contributes to obesity-associated BC progression [13].

The renin–angiotensin system (RAS) is classically known to regulate fluid balance and blood pressure. Angiotensinogen (Agt) is the main precursor protein for the bioactive hormone angiotensin II (Ang II). Agt is first cleaved by renin into Ang I, which is converted into Ang II by the angiotensin-converting enzyme (ACE). Ang II mediates its effect via binding to two major G-protein-coupled receptors, namely, type 1 receptor (AT1R) and type 2 receptor (AT2R) [14,15]. Adipose RAS activation has multiple roles in obesity, such as increasing inflammatory, prothrombotic, and angiogenic markers [16] that can also promote tumor growth, proliferation, and invasion in the breast [17,18]. In addition, Ang II/AT1R signaling triggers production and infiltration of tumor-associated macrophages (TAM) in different tumor models, while RAS inhibitors can restrain tumor growth and TAM response [19]. Thus, RAS is a potential key player in adipocyte–breast-cancer-cell interactions. RAS inhibitors such as angiotensin-converting enzyme inhibitors (ACE-I) and AT1R blockers (ARB) have recently gained interest as possible anticancer agents in both clinical and preclinical models of BC [17,18,20,21]. Some ACE-I and ARBs were found to reduce BC risk in a time-dependent manner in BC patients [21].

RAS can also be downregulated by anti-inflammatory dietary bioactive compounds such as omega-3 polyunsaturated fatty acids (n-3 PUFA) [22], which we have also shown to reduce Agt secretion from adipocytes [23]. The dietary n-3 PUFAs eicosapentaenoic acid (EPA)-20:5n-3 and docosahexaenoic acid (DHA)-22:5n-3 have potent anti-inflammatory properties that include reducing obesity-associated systemic and adipose tissue inflammation, thus alleviating TME formation in adipocyte–BC-cell crosstalk [24,25,26,27]. Therefore, given the promising anti-inflammatory and anticancer properties of ACE-I and EPA, we hypothesized that ACE-I (captopril (CAP)) and EPA alone or in combination would attenuate BC cell inflammation and migration, in part through inhibition of adipocyte RAS. We examined both direct and adipose CM-mediated effects of captopril and EPA and their combination on cultured BC cell genes and proteins as well as cell migration using wound healing analyses.

## 2. Results

### 2.1. Effect of Captopril and EPA on Markers of Fatty Acid Synthesis and Inflammation in BC Cells and Role of Human Adipocyte-Conditioned Media (CM)

**Figure 1:**
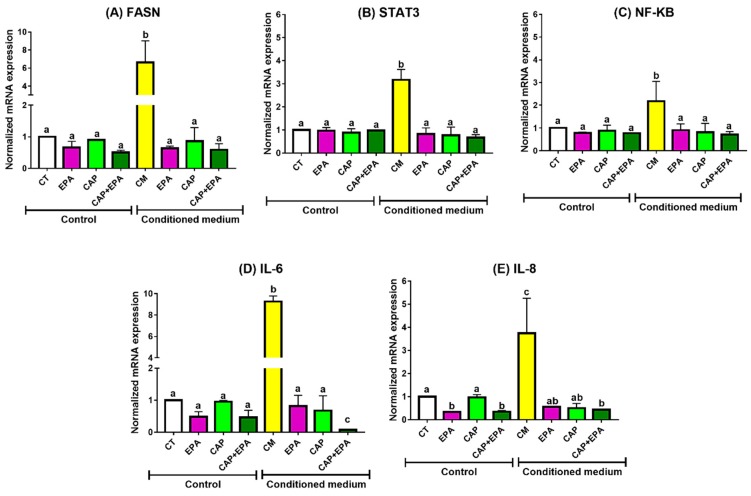
Eicosapentaenoic acid (EPA) and captopril (CAP) (angiotensin-converting enzyme inhibitors; ACE-I) effects on mRNA expression in MDA-MB-231 cells. MDA-MB-231 cells were treated with 100 µm of CAP with or without 100 µm of EPA for 48 h. Human mesenchymal stem cells (HMSCs) were differentiated into adipocytes and treated with 100 µm of CAP with or without 100 µm of EPA for 24 h. Conditioned media (CM) was collected and transferred to breast cancer (BC) cells for 48 h. Cells were harvested and changes in mRNA levels of fatty acid synthase (FASN) (**A**), signal transducer and activator of transcription 3 (STAT3) (**B**), nuclear factor kappa B (NF-κB) (**C**), interleukin (IL)-6 (**D**), and IL-8 (**E**) were measured (*p* < 0.05; N = 3; three replicates under each treatment group; bars with different letters (a, b, c) indicate significance).

MDA-MB-231 and MCF-7 cells were treated with 100 µm of EPA or 100 µm of CAP with or without EPA either directly or using CM from CAP, EPA, or both pretreated adipocytes for 48 h. Direct treatments with EPA, CAP, or CAP + EPA did not alter most measured markers of cancer cell growth and inflammation in MDA-MB-231 cells, with the exception of IL-8, which was significantly reduced by EPA and CAP + EPA treatments for 48 h in MDA-MB-231 cells (*p* < 0.05) compared with control (CT), while CAP alone had no effect. However, EPA and CAP + EPA had comparable effects, indicating no additional effect of direct CAP + EPA combination on BC cell inflammatory markers (Figure 1E). Treatment of MDA-MB-231 cells with human adipocyte CM significantly increased all tested markers of cell growth and inflammation after 48 h, as shown by increased mRNA levels of FASN, STAT3, NF-κB, IL-6, and IL-8 compared with control (Figure 1A–E; also see Tables S1–S7) (*p* < 0.05). However, treatment of MDA-MB-231 cells with CM from adipocytes pretreated with EPA, CAP, and their combination significantly reduced the mRNA content of all measured markers of BC cell growth and inflammation compared with treatment with CM derived from untreated adipocytes (Figure 1A–E) (*p* < 0.05). However, no changes were observed in FASN, STAT3, NF-κB, and IL-8 mRNA transcription levels in MDA-MB-231 BC cells treated with CAP + EPA pretreated adipocyte CM, compared with CAP-CM or EPA-CM (Figure 1A–C,E) (*p* < 0.05). Interestingly, CAP + EPA pretreated CM reduced IL-6 mRNA levels to a greater extent in MDA-MB-231 cells compared with CAP-CM and/or EPA-CM treatments, indicating potential additive anti-inflammatory effects of CAP and EPA combination (Figure 1D) (*p* < 0.05). Exploratory factorial regression analyses performed to examine the interactions of CAP and EPA when administered as a combination resulted in significant negative regression coefficients for CM-CAP and CM-EPA factors but significant positive CM-CAP × EPA interaction for mRNA levels of all measured markers of MDA-MB-231 cell growth and inflammation (Tables S1–S5). This result suggests that CAP and EPA may act via a common pathway in reducing mRNA expression in CM-treated MDA-MB-231 cells.

**Figure 2:**
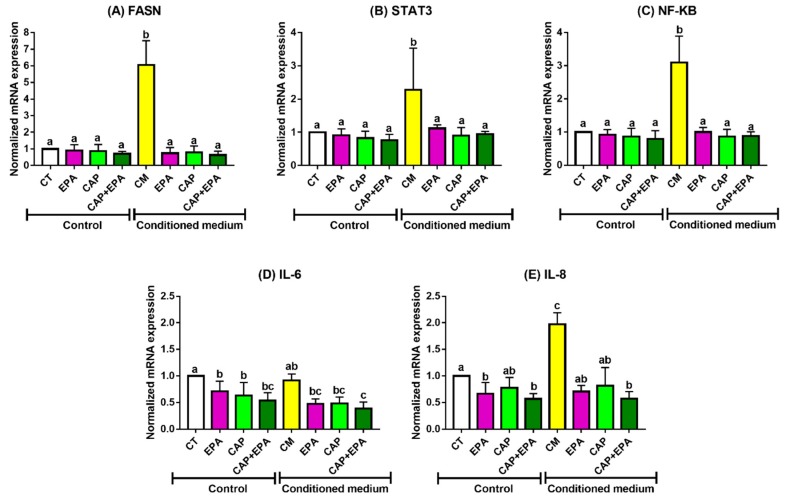
EPA and captopril (ACE-I) effects on mRNA expression in MCF-7 cells. MCF-7 cells were treated with 100 µm of CAP with or without 100 µm of EPA for 48 h. HMSCs were differentiated into adipocytes and treated with 100 µm of CAP with or without 100 µm of EPA for 24 h. CM was collected and transferred to BC cells for 48 h. Cells were harvested and mRNA level changes of FASN (**A**), STAT3 (**B**), NF-κB (**C**), IL-6 (**D**), and IL-8 (**E**) were measured (*p* < 0.05; N = 3; three replicates under each treatment group; bars with different letters (a, b, c) indicate significance).

On the other hand, CM from human adipocytes significantly increased markers of cell growth and inflammation in MCF-7 cells after 48 h, as shown by increased mRNA levels of FASN, STAT3, NF-κB, and IL-8 compared with CT (*p* < 0.05), while CM from adipocytes pretreated with EPA, CAP, and CAP + EPA significantly reduced the abovementioned markers of cell growth and inflammation after 48 h compared with CM-control (Figure 2A–C,E; Tables S8–S14) (*p* < 0.05). However, no changes in the mRNA levels of the respective markers were observed between CAP and EPA treated groups with or without CAP–EPA combination. Additionally, direct treatments with EPA and CAP + EPA significantly reduced MCF-7 BC cell inflammation, as demonstrated by significantly lower IL-6 and IL-8 mRNA transcription levels, while direct treatments with CAP reduced only IL-6 mRNA levels after 48 h compared with control in MCF-7 cells (Figure 2D,E) (*p* < 0.05). However, the changes were not significant between EPA and CAP + EPA treated groups, indicating no additional effects of CAP–EPA combination in MCF-7 cells compared with EPA alone or CAP alone.

**Figure 3:**
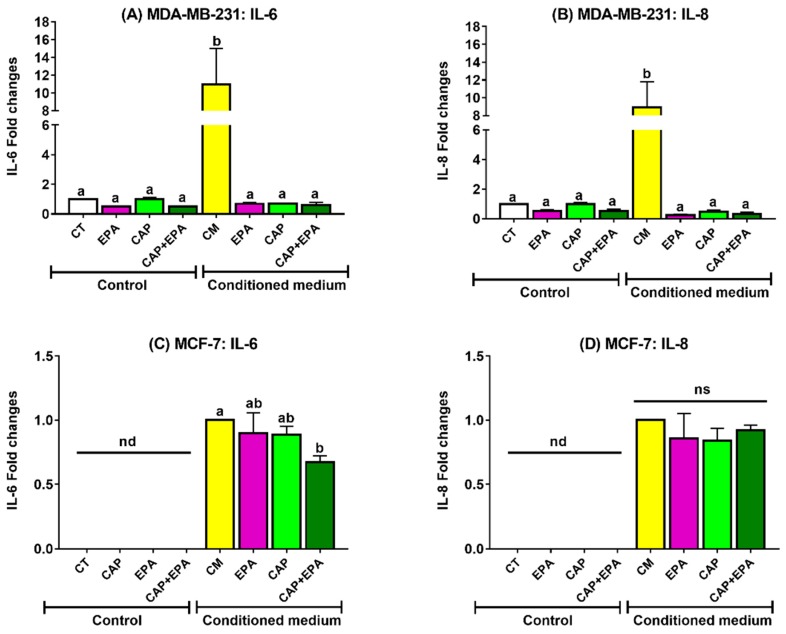
Adipose-CM with ACE-I with or without EPA reduced proinflammatory interleukins secretion in BC cells. Mature human adipocytes were pretreated with 100 µM of CAP with or without 100 µm of EPA for 24 h followed by CM collection and transferred to MDA-MB-231 (**A**,**B**) and MCF-7 (**C**,**D**) cells for 48 h. Medium was collected, and secreted IL-6 and IL-8 levels were measured in response to direct (no adipocyte CM) or CM-mediated CAP and EPA effects (*p* < 0.05; N = 3; and in each experiment, three replicates were used for each treatment group; bars with different letters (a, b) indicate significance;); nd: not detectable; ns: not significant).

Next, we measured proinflammatory IL-6 and IL-8 protein levels secreted by both MDA-MB-231 and MCF-7 cells after 48 h of EPA and CAP treatments. Medium was collected from both cell lines after direct and adipocyte CM (with or without EPA, CAP, or combined EPA and CAP) treatments for 48 h. Consistent with the above-described results (Figure 3A,B), no changes were observed in secreted IL-6 and IL-8 levels in MDA-MB-231 cells with direct treatments of EPA, CAP, CAP + EPA for 48 h. In addition, increased IL-6 and IL-8 secretion in MDA-MB-231 cells were observed in response to adipocyte CM treatments compared with CT and direct CAP ± EPA treatments (*p* < 0.05). Moreover, significant reductions in both IL-6 and IL-8 protein levels were identified in MDA-MB-231 cells in response to CM-EPA and CM-CAP treatments (with or without EPA) compared with CM-control (*p* < 0.05) (Figure 3A,B). However, the changes were not significant between CM-EPA and CM-CAP groups as well as compared to CT and direct CAP ± EPA treatments after 48 h (*p* < 0.05). Additionally, both IL-6 and IL-8 protein levels were undetectable (* nd) without CM treatments in MCF-7 cells, as reported earlier by others [28] (Figure 3C,D). Interestingly, adipocyte CM with CAP + EPA significantly reduced IL-6 secretion in MCF-7 cells compared with CM-control treatment, but no changes were observed compared to CM-EPA or CM-CAP individual treatments (Figure 3C) (*p* < 0.05). However, no significant changes were observed in secreted IL-8 levels by MCF-7 cells when treated with adipocyte CM-EPA or CM-CAP (with or without EPA) (Figure 3D). Therefore, these results suggest possible inflammatory-reducing benefits of CAP–EPA combination in triple-negative breast cancer (TNBC) MDA-MB-231 cells but not in MCF-7 cells, indicating the potential role of n-3 PUFAs and antihypertensive ACE inhibitors in attenuating the tumor-promoting proinflammatory effects of adipocytes on breast cancer cells.

### 2.2. Combined Effect of Captopril and EPA on Breast Cancer Cell Migration in Response to Treatment with Adipocyte CM, Measured by a Wound Healing Assay

**Figure 4:**
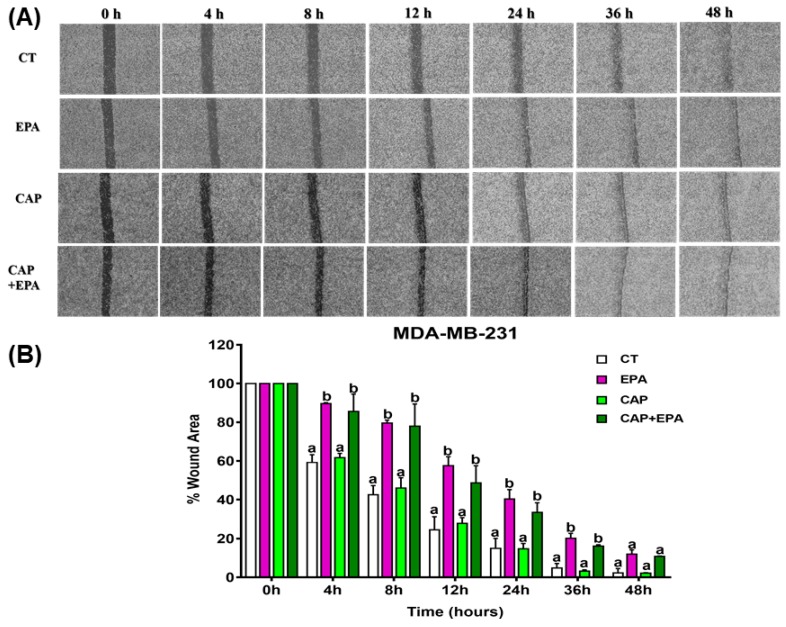
Direct effect of captopril and EPA on cell migration in MDA-MB-231 cells. Representative wound healing images at 0, 4, 8, 12, 24, 36, and 48 h. Wounds were inflicted with a 200 uL pipette tip on MDA-MB-231 (**A**,**B**) cells in confluent monolayers. Graphical representation of migrating MDA-MB-231 (**A**,**B**) cells in response to CAP with or without EPA treatments of BC cells. Cells treated with regular Dulbecco’s Modified Eagle’s Medium (DMEM) with 1% bovine serum albumin (BSA) conjugation were used as controls (BSA). Bars represent mean of the percent wound area in MDA-MB-231 (**A**,**B**) cells in the two independent experiments ± SEM. *p* < 0.05 (comparison between 0 and 48 h; N = 2 combined experiments, in which each had three replicates per treatment group). Different letters (a, b) indicate significance.

Since inflammation is associated with BC cell migration, we performed in vitro wound healing assays to dissect the effects of both direct and human adipocyte-mediated effects of CAP ± EPA treatments in MDA-MB-231 cells. First, MDA-MB-231 cells were treated directly with CAP with or without EPA and images of wound closure were taken at various times up to 48 h (Figure 4A,B; also see Tables S15 and S16). No significant effects of direct CAP treatment were found in MDA-MB-231 cells at any of the time points tested (*p* < 0.05) (Figure 4A,B). Intriguingly, a significantly higher percent wound area (or lower percent wound healing) was observed in response to direct EPA and CAP + EPA treatments at 4, 8, 12, 24, and 36 h time points when compared with bovine serum albumin (BSA)-control (*p* < 0.05), indicating reduced wound healing cell migration with EPA and CAP + EPA. However, the reductions in wound healing were not significant between EPA and CAP + EPA treatments at the respective time points (*p* < 0.05), indicating no additional effect of CAP when combined with EPA on MDA-MB-231 cell wound-healing capacity (Figure 4A,B).

**Figure 5:**
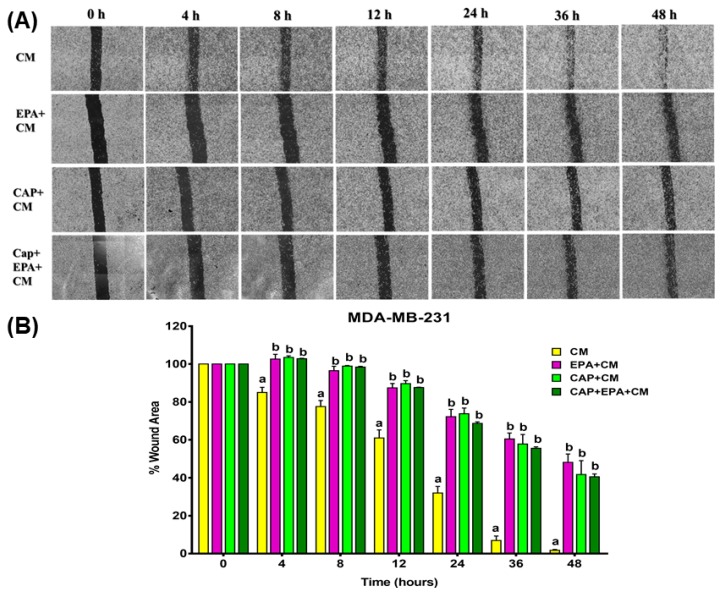
CM-mediated effect of captopril and EPA on cell migration in MDA-MB-231 cells. Representative wound healing images at 0, 4, 8, 12, 24, 36, and 48 h. Wounds were inflicted with a 200 uL pipette tip on MDA-MB-231 (**A**,**B**) cells in confluent monolayers. Graphical representation of migrating MDA-MB-231 (**A**,**B**) cells in response to CM-EPA and CM-CAP with or without EPA treatments of BC cells. Cells treated with human adipose conditioned medium were used as controls (CM). Bars represent mean of the percent wound area in MDA-MB-231 (**A**,**B**) cells in the two independent experiments ± SEM. *p* < 0.05 (comparison between 0 and 48 h; N = combined experiments, in which each had three replicates per treatment group). Different letters (a, b) indicate significance.

Next, we identified the adipocyte-CM-mediated effects of CAP ± EPA treatments in MDA-MB-231 cells. Mature human adipocytes were pretreated with CAP with or without EPA for 24 h and medium was transferred to MDA-MB-231 cells, followed by capturing time-dependent images, as described above (Figure 5A,B). Cell migration, as denoted by the changes in percent wound area due to closure, was significantly increased in response to CM-mediated EPA, CAP, and CAP + EPA treatments compared with CM-control in MDA-MB-231 cells at all time points tested, such as 4, 8, 12, 24, 36, and 48 h (*p* < 0.05) (Figure 5A,B), whereas CM-CAP + EPA effects were not significantly different from CM-EPA or CM-CAP, indicating no additional effect of CAP and EPA combination on MDA-MB-231 cell migration (Figure 5A,B). When time was modeled as a continuous variable, the wound area with CM treatment decreased by 2.13% per hour. This rate of reduction of wound area was hindered by treatment of adipocyte CM with each of EPA, CAP, and their combination as evidenced by significant positive interactions (Table S16). The subsequent factorial model revealed a significant positive effect of EPA and CAP but a significant negative interaction of CAP and EPA; these findings suggest that EPA and CAP may act via a common pathway in slowing down cell MDA-MB-231 cell migration (Tables S17 and S18). We did not perform wound healing assays in MCF-7 cells since the inflammatory markers were expressed and secreted at a lower level in MCF-7 compared with the MDA-MB-231 cell line we used. In addition, we observed greater changes in these markers, such as IL-6 in MDA-MB-231 cells, in response to both direct and CM-mediated treatments of CAP and EPA. These results further confirm our previous observation that CAP and EPA effects may be mediated by a common pathway to reduce expression of mRNA biomarkers of MDA-MB-231 cell proliferation.

## 3. Discussion

The aim of the current study was to provide new insights into adipocyte–BC interaction through inhibition of RAS in adipocytes. We previously showed protective effects of n-3 PUFAs in adipocyte–BC cell interactions [27]. Moreover, we demonstrated that n-3 PUFAs reduce Agt secretion from adipocytes and reduce both systemic and adipose tissue inflammation [29]. Hence, we proposed to determine whether an n-3 PUFA (EPA), individually or in combination with ACE-I, would reduce BC cell inflammation and migration directly on BC cells or via adipose RAS inhibition. In this study, we demonstrated reduced BC cell inflammation and motility in response to CM-derived EPA, CAP, and CAP + EPA treatments while no changes were observed among these treatments, indicating that the combination of CAP and EPA had no synergistic effects in attenuating adipocyte RAS effects on BC cells.

The doses of EPA and CAP that we used were within or lower than ranges used in human studies, making our findings translatable to humans. For omega-3 fatty acids, doses ranging from 1 to 6 g of EPA per day have been used in various human cancer studies and are associated with both improved cardiovascular and cancer outcomes [30,31]. Researchers reported that a ≥150 µg/mL (~150 µm) plasma EPA level was linked to benefits in preventing cardiovascular disease (CVD) outcomes [32]. In addition, plasma EPA concentrations in healthy subjects taking 2–4 g/day of EPA ethyl ester for 28 days increased up to a mean of 366 µg/mL, without any adverse events [33]. Although it is difficult to translate in vitro doses into actual human intake, the above mentioned reports clearly indicate that our 100 µm of EPA dose was lower than doses that can be reached in human plasma levels with prescribed fish oil or EPA. Moreover, other cell studies used doses of fatty acids up to 5 times higher than our dose [34,35]. For captopril, it has been used safely at doses up to 450 mg/day in humans [36]**,** which may lead to plasma doses likely higher than the 100 µm dose we used for cell treatments. Captopril was given at 150 mg/day to lung cancer patients [37] and lisinopril (another ACE-I) given as 10 mg tablets to breast cancer patients to prevent cardiotoxicity [38]. We were unable to find data regarding this drug’s plasma level concentration in cancer patients since ACE-I is only prescribed as antihypertensive medication for cancer patients and not as an anticancer agent. Hence, the suitable dose for preventing breast cancer is not known. However, earlier research reported plasma captopril levels in normal versus chronic renal failure subjects as 364 and 347 µm, respectively, after 1 h, but it was undetectable in blood after 6 h [39]. In addition, we previously performed a cell viability (MTT) assay with both 100 µm of EPA and 100 µm of captopril and found no toxic effects against breast cancer cell viability.

Adipose tissue is a major component of the breast tissue and is actively involved in forming TME; thus, adipose tissue through its inflammatory and lipogenic activities may increase the risk of BC development [3,40]. Human adipose CM is a source of various growth factors, several adipocyte secretory hormones, cytokines, and metabolites [41], which are immensely responsible for inducing expression of markers associated with breast cancer cell signaling, motility, and metastases [42,43]. Consistent with this, we have found increased inflammatory, IL-6, IL-8, NF-κB, and STAT3 mRNA expression as well as induced expression of the lipogenic enzyme FASN in both MCF-7 and MDA-MB-231 cells in response to human adipocyte CM. Overexpression of FASN plays an important role in tumorigenesis and is thus a vital target of obesity-mediated BC therapy [44]. Additionally, selective FASN inhibitors are potent antitumor agents showing antiproliferative and apoptosis inhibition effects in in vitro and in vivo models of receptor-positive (ER/HER2+) BC [45].

Moreover, two metabolically different BC cell lines, MCF-7 and MDA-MB-231, were used in the current study to identify the presence of any metabolic responses of these cell lines to n-3 PUFA or ACE-I. While the observed changes in mRNA levels between two cell lines were almost identical, their cytokine secretory profiles varied in response to direct or CM treatments. Both IL-6 and IL-8 levels were below detection in response to direct EPA/CAP treatments in MCF-7 cells, which is in agreement with other studies investigating the basal protein secretion levels by different BC cell lines [46,47]. Furthermore, we observed higher IL-6/IL-8 secretion by MDA-MB-231 cells compared with MCF-7 cells, which is consistent with previous studies indicating a stronger secretory profile in MDA-MB-231 TNBC cells compared with its less aggressive counterpart MCF-7 cells [48]. Another possible reason for the differential proinflammatory profile of MCF-7 versus MDA-MB-231 could be the intrinsically reduced transcriptional/post-transcriptional and/or translational/post-translational IL-6/IL-8 profile of MCF-7 cells in vitro compared with MDA-MB-231 cells [48].

Moreover, IL-6 activates STAT3 and NF-κB by (i) autocrine and paracrine signaling [8] and (ii) via synergistic crosstalk between the pathways in BC cells [7], resulting in cancer cell proliferation and apoptosis evasion [8]. By contrast, EPA is a natural anti-inflammatory bioactive compound previously reported to reduce IL-6 production, in part by inhibiting NF-κB activation [49] and through its downstream anti-inflammatory lipid mediators such as E-resolvins [50]. Consistent with this, we have also shown reduced mRNA levels of IL-6/STAT3/NF-κB in response to EPA-CM treatments. On the other hand, EPA also reduces both inflammation and Agt secretion from adipocytes both in vivo and in vitro [22,29]. Hence, when combining EPA with ACE-I (captopril), we observed reduced IL-6 inflammation in both MDA-MB-231 and MCF-7 BC cells as indicated by lower IL-6 mRNA and protein levels, respectively. Interestingly, the effect of EPA and ACE-I combination was more pronounced in reducing TNBC cell inflammation as shown by reduced IL-6 levels in MDA-MB-231 cells, which could be attributed to the more aggressive, inflammatory, and metastatic nature of MDA-MB-231 cells compared with MCF-7 cells [51]. Similarly, EPA and EPA-derived ethanol amines were more potent in reducing cell proliferation, migration, and invasion of MDA-MB-231 cells compared with non-invasive MCF-7 cells in vitro, which is in agreement with our present study [52]. We did not observe any synergistic effects of ACE-I and EPA combination in BC cell proliferation and migration, possibly due to their competitive anti-inflammatory in vitro mechanisms of action via EGFR and/or NF-κB (Rel-A) signaling pathways [19,53]. Moreover, many preclinical studies reported antihypertensive and anti-inflammatory roles of n-3 PUFAs respectively via ACE-2 upregulation, Ang II downregulation, and partly via GPCR-mediated NF-κB modulation [22,54,55]. Importantly, to our knowledge, we are the first to report the potential anti-inflammatory roles of ACE-I and EPA combination in targeting adipocyte and BC cell interactions. Despite this knowledge, potential mechanisms of ACE-I and EPA interactions in lessening the effects of adipocytes on BC cells remain unknown. Clinical studies are also lacking to understand the crosstalk between RAS inhibitors and n-3 PUFAs or other anti-inflammatory bioactive compounds to improve obesity-mediated cancer outcomes.

Inflammation is positively associated with BC cell motility and wound healing, although their mechanistic inter-relationship requires further studies [56]. Adipose CM contains various cytokines, including IL-6 and IL-8, which could be responsible for increased BC cell motility and wound healing, as reported by others [57]. We found a similar induced wound-healing capacity in MDA-MB-231 cells in response to human adipose CM. On the contrary, given the anti-inflammatory and antiproliferative effects of EPA on MDA-MB-231 cell motility [27] and the proposed similar effects for ACE-I, we combined them to identify their synergistic or additive effects in MDA-MB-231 TNBC. Surprisingly, both direct and CM-mediated EPA and ACE-I combination were ineffective in reducing TNBC cell motility compared with either EPA or ACE-I alone. This is in disagreement with Krusche et al., who reported the synergistic inhibitory effect of ACE-I and a Chinese herb (artesunate) on human umbilical vein endothelial cells (HUVEC) motility [58]. This might be due to the competitiveness between EPA and captopril to target common inflammatory mechanisms of action such as NF-κB downregulation and/or inactivation [59]. Taken together, despite some protective anti-inflammatory effects of EPA and ACE-I combination, such as attenuating IL-6, the underlying mechanisms require further studies in both in vitro and in vivo models of obesity-induced breast cancer.

## 4. Materials and Methods

### 4.1. Cell Culture Experiments

Human breast cancer cells such as MDA-MB-231 (HTB-26; Lot: 700792) TNBC and ER/PR positive MCF-7 (HTB-22; Lot: 64125078) BC cells were purchased from the American Type Culture collection (ATCC) (Manassas, VA, USA). MDA-MB-231 and MCF-7 cells were seeded at a density of 50,000 cells per well and maintained under standard culture conditions at 37 °C in a humid atmosphere of 5% CO_2_ in Dulbecco’s Modified Eagle’s Medium (DMEM). The medium was supplemented with 10% fetal bovine serum (FBS) (Thermo Fisher Scientific, Waltham, MA, USA) and antibiotics (50 µg/mL penicillin, 50 µg/mL streptomycin, and 100 ug/mL neomycin) (Thermo Fisher Scientific, Waltham, MA, USA). Bone-marrow-derived human mesenchymal stem cells (HMSCs) (PT-2501; Lot: 0000483199) were purchased from Lonza (Allendale, NJ, USA). These cells were differentiated into mature human adipocytes according to the optimized published protocol [60] and maintained under standard culture conditions at 37 °C and 5% CO_2_ in DMEM: Nutrient Mixture F-12 (DMEM/F-12) (Thermo Fisher Scientific, Waltham, MA USA). Following differentiation, cells were transferred to regular DMEM with 10% FBS for 24 h and CM experiments were performed. EPA (Nu-Check Prep, Waterville, MN, USA) and/or captopril (Sigma-Aldrich, St. Louis, MO, USA) was conjugated with 1% BSA (Sigma-Aldrich, St. Louis, MO, USA) for 2 h in a shaking water bath at 37 °C prior to cell treatment. Since fatty acids are found in the circulation as attached to albumin, many studies used BSA as a complex medium for EPA and other fatty acids in in vitro culture experiments [61]. For consistency, all treatments were performed in a BSA complexed medium.

### 4.2. Treatment with ACE Inhibitor, Captopril, and Eicosapentaenoic Acid for Conditioned Medium Experiments

MDA-MB-231 and MCF-7 cells were starved with 1% BSA for 2 h prior to CAP ± EPA experiments. The BC cells were treated directly with the ACE inhibitor CAP (100 µm) with or without EPA (100 µm) combination for 48 h to identify their individual/combined effect in modulating BC cell metabolism (Figure S1). The time and dose of our current treatments (CAP and EPA) were based on our previous work using EPA alone [27] or CAP alone (manuscript under review) against adipocyte–BC-cell interactions. CM experiments were performed between differentiated human adipocytes (HMSCs) and BC cells. Prior to the treatments, differentiated mature adipocytes were starved with 1% BSA for 2 h. Following starvation, mature adipocytes were treated with 100 µm of CAP ± 100 µm of EPA complexed with BSA, and BSA alone was used as the control. Conditioned media from differentiated adipocytes were collected after 24 h and centrifuged at 10,000× *g* for 10 min, then filter-sterilized to remove any cell debris. MDA-MB-231 and MCF-7 cells were seeded in six-well plates and then at 70% confluency exposed to CM from human adipocytes for 48 h. BC cells and media were collected and stored at −80 °C for further analyses (Figure S1).

### 4.3. Enzyme-Linked Immunosorbent Assay (ELISA)

Quantification of secreted IL-6 and IL-8 levels were determined by ELISA (R&D Systems, Minneapolis, MN, USA) according to the manufacturer’s protocol.

### 4.4. RNA Isolation and Real-Time Quatitative Polymerase Chain Reaction (RT-qPCR)

RNA was purified using the Quick RNA mini kit (Zymo Research, Irvine, CA, USA) according to the established manufacturer’s protocol followed by cDNA synthesis. cDNA was reverse-transcribed using Maxima reverse transcriptase (Thermo Fisher Scientific, Waltham, MA, USA). mRNA transcription levels were assessed with RT-qPCR using the Sybr green master mix (Thermo Fisher Scientific, Waltham, MA, USA). All genes were normalized to two housekeeping genes (18S ribosomal RNA and TBP (TATA box binding protein)). We used both 18S and TBP as our reference genes for normalization of RT-qPCR data, both were normalized to each other, and we did not see any regulatory effects in response to our proposed treatment conditions.

Sequences of the primers (Sigma Aldrich, St. Louis, MO, USA) are listed as follows (forward, reverse):

IL-6 (5′-AGACAGCCACTCACCTCTTCAG-3′, 5′-TTTCTGCCAGTGCCTCTTTGC-3′),
IL-8 (5′-AGGACAAGAGCCAGGAAGAA-3′, 5′-GGGTGGAAAGGTTTGGAGTATG-3′),
NF-κB (5′-ATGGCTTCTATGAGGCTGAG-3′, 5′-GTTGTTGTTGGTCTGGATGC-3′),
STAT3 (5′-AGAAGGACATCAGCGGTAAGA-3′, 5′-GGATAGAGATAGACCAGTGGAGAC-3′),
FASN (5′-TCGTGGGCTACAGCATGGT-3′, 5′-GCCCTCTGAAGTCGAAGAAGAA-3X),
18S (5′-CTACCACATCCAAGGAAGCA-3′, 5’-TTTTTCGTCACTACCTCCCCG-3′), and
TBP (5′-ATGGTGGTGTTGTGAGAAGATG-3′, 5′-CAGATAGCAGCACGGTATGAG-3′).

### 4.5. Wound Healing Assay

MDA-MB-231 cells were seeded (50,000 cells per well) in six-well plates. At 95% confluence, cells were washed with phosphate-buffered saline (PBS) (Thermo Fisher Scientific, Waltham, MA, USA) and starved with 0.5% FBS in DMEM overnight to inhibit cell proliferation, thereby ensuring wound closure was due to cell migration only. Using a sterilized 200 μL pipette tip, two straight scratches were made through the cell’s monolayer, stimulating wound. Cells were first rinsed once gently with PBS, then subjected to treatments described in the results, such as CAP with or without EPA. Images were taken at 0, 4, 8, 12, 24, 36, and 48 h using the EVOS Cell Imaging System (Thermo Fisher Scientific, Waltham, MA, USA) and data were analyzed using the in-house Cellular Growth Analyzer for Windows software (version 2.2). The in-house analyzer program was used to change the original image into black (the scratch) and the cell area into white (inside cells). The detection parameter assigned in the program was the pixel size, and the program checked along the *x* direction and later along the *y* direction, moving from left to right and counting the number of black pixels it came across. This stopped when it encountered a white pixel. This worked on the basis that the wound area (black) was the largest gap visible in the scratch assay.

### 4.6. Statistical Analysis

Data obtained from the experiments were normalized and analyzed using IBM SPSS (version 20, 2015) and R statistical software (version 3.5.3), and graphs were made using GraphPad Prism (Version 8.0). Statistical significance between groups (control vs. treatments) was tested using one-way ANOVA and subsequent pairwise post hoc comparisons adjusted using Tukey’s correction. Differences were considered statistically significant for a Tukey-corrected *p*-value < 0.05. All experiments were performed at least two times and results are expressed as mean ± SEM of three independent biological replicates. Additional exploratory factorial regression analyses were performed to examine the interactions between EPA and CAP on all measured biomarkers.

For the wound healing assays, the data were analyzed separately for BSA and CM-treated conditions using two-way ANOVA models. The two-way ANOVA models were constructed to examine for the main effects of treatment (i.e., EPA, CAP, and combination), time modeled as a categorical variable, and their interaction. To determine the effect of each treatment on each biomarker per unit time, additional exploratory linear regression analyses were performed to modeling time as a continuous variable and the treatment condition as a categorical variable and their interaction. The interactions of EPA and CAP on temporal trends of biomarkers were examined in factorial regression analyses.

## 5. Conclusions

In conclusion, our study reported significant protective roles of EPA with and without ACE-I combination in attenuating adipocyte-induced proinflammation and cancer cell migration. To our knowledge, our study is the first to report inflammatory IL-6 downregulation effects of captopril and EPA combination in both MDA-MB-231 and MCF-7 BC cells. One acknowledged limitation of our study is that we only established a proof of principle for protective effects of CAP–EPA combination against adipocyte–BC-cell interaction, possibly via modulation of inflammation. This provided us a basis for future more in-depth mechanistic studies to further dissect effects of adipocytes on cancer cell proliferation and motility in response to CAP and EPA treatments in the tumor microenvironment.

Additionally, we did not see any differences between individual and combined ACE-I and EPA treatments except for IL-6. This indicates the need for more in-depth mechanistic studies possibly via knockdown of inflammatory signaling pathways in adipocytes and BC cells such as RAS and IL-6/STAT3/NF-κB pathways. Moreover, comparison of the efficacy of other ACE-I such as lisinopril, ARBs such as telmisartan, losartan, and/or n-3 PUFAs such as DHA or fish oil is necessary to better understand how these affect adipocyte–BC-cell interactions. Hence, our present study provides a novel significant validation for future mechanistic studies to combine diet and antihypertensive medication as a potential therapeutic approach for obesity-associated BC. More research is warranted to identify the possible mechanism of action and synergy of this unique combination of ACE-I and EPA or other n-3 PUFAs in obesity and breast cancer.

## Supplementary Materials

The following are available online at https://www.mdpi.com/2072-6694/12/1/220/s1, Figure S1: Experimental design; Table S1: Exploratory factorial regression analyses to examine CAP and EPA interactions of mRNA markers associated with MDA-MB-231 cell growth and inflammation; Table S2: Exploratory factorial regression analyses to examine CAP and EPA interactions of mRNA markers associated with MDA-MB-231 cell growth and inflammation; Table S3: Exploratory factorial regression analyses to examine CAP and EPA interactions of mRNA markers associated with MDA-MB-231 cell growth and inflammation; Table S4: Exploratory factorial regression analyses to examine CAP and EPA interactions of mRNA markers associated with MDA-MB-231 cell growth and inflammation; Table S5: Exploratory factorial regression analyses to examine CAP and EPA interactions of mRNA markers associated with MDA-MB-231 cell growth and inflammation; Table S6: Exploratory factorial regression analyses to examine CAP and EPA interactions of protein markers associated with MDA-MB-231 cell inflammation; Table S7: Exploratory factorial regression analyses to examine CAP and EPA interactions of protein markers associated with MDA-MB-231 cell inflammation; Table S8: Exploratory factorial regression analyses to examine CAP and EPA interactions of mRNA markers associated with MCF-7 cell growth and inflammation; Table S9: Exploratory factorial regression analyses to examine CAP and EPA interactions of mRNA markers associated with MCF-7 cell growth and inflammation; Table S10: Exploratory factorial regression analyses to examine CAP and EPA interactions of mRNA markers associated with MCF-7 cell growth and inflammation; Table S11: Exploratory factorial regression analyses to examine CAP and EPA interactions of mRNA markers associated with MCF-7 cell growth and inflammation; Table S12: Exploratory factorial regression analyses to examine CAP and EPA interactions of mRNA markers associated with MCF-7 cell growth and inflammation; Table S13: Exploratory factorial regression analyses to examine CAP and EPA interactions of protein markers associated with MCF-7 cell inflammation; Table S14: Exploratory factorial regression analyses to examine CAP and EPA interactions of protein markers associated with MCF-7 cell inflammation; Table S15: Exploratory time-series regression analyses to examine CAP and EPA interactions in MDA-MB-231 cell migration; Table S16: Exploratory time-series regression analyses to examine CAP and EPA interactions in MDA-MB-231 cell migration; Table S17: Exploratory time-series factorial regression analyses to examine CAP and EPA interactions in MDA-MB-231 cell migration; Table S18: Exploratory time-series factorial regression analyses to examine CAP and EPA interactions in MDA-MB-231 cell migration.

## Abbreviations

Agt	Angiotensinogen
Ang I	Angiotensin I
Ang II	Angiotensin II
ACE	Angiotensin-converting enzyme
ACE-I	Angiotensin-converting enzyme inhibitor
ARB	Angiotensin receptor type I blocker
AT1R	Angiotensin receptor type I
AT2R	Angiotensin receptor type II
ATCC	American Type Culture Collection
BC	Breast cancer
CAA	Cancer-associated adipocytes
CAP	Captopril
CM	Conditioned medium
CVD	Cardiovascular disease
DHA	Docosahexaenoic acid
DMEM	Dulbecco’s Modified Eagle’s Medium
ELISA	Enzyme-linked immunosorbent assay
EPA	Eicosapentaenoic acid
FBS	Fetal bovine serum
FASN	Fatty acid synthase
ER	Estrogen receptor
ER+	Estrogen receptor positive
ER-	Estrogen receptor negative
GAPDH	Glyceraldehyde-3-phosphate dehydrogenase
HER2+	Human epidermal growth factor receptor-2 positive
HMSC	Human mesenchymal stem cells
HUVEC	Human umbilical vein endothelial cells
IL-6	Interleukin-6
IL-8	Interleukin-8
MCP-1	Monocyte chemoattractant protein 1
MMP	Matrix metalloproteinase
n-3 PUFA	Omega-3 polyunsaturated fatty acid
NF-κB	Nuclear factor kappa B
PBS	Phosphate-buffered saline
PR	Progesterone receptor
RAS	Renin–angiotensin system
RT-qPCR	Real-time quantitative polymerase chain reaction
STAT3	Signal transducer and activator of transcription 3
TAM	Tumor-associated macrophage
TME	Tumor microenvironment
TNBC	Triple-negative breast cancer
VEGF	Vascular endothelial growth factor

## References

[1] Stewart B., Wild C.P. (2017). World Cancer Report 2014.

[2] Carmichael A.R. (2006). Obesity as a risk factor for development and poor prognosis of breast cancer. BJOG Int. J. Obstet. Gynaecol..

[3] Ahn J., Schatzkin A., Lacey J.V., Albanes D., Ballard-Barbash R., Adams K.F., Kipnis V., Mouw T., Hollenbeck A.R., Leitzmann M.F. (2007). Adiposity, Adult Weight Change, and Postmenopausal Breast Cancer Risk. Arch. Intern. Med..

[4] Simone V., D’avenia M., Argentiero A., Felici C., Rizzo F.M., De Pergola G., Silvestris F. (2016). Obesity and breast cancer: Molecular interconnections and potential clinical applications. Oncologist.

[5] Deng T., Lyon C.J., Bergin S., Caligiuri M.A., Hsueh W.A. (2016). Obesity, Inflammation, and Cancer. Annu. Rev. Pathol. Mech. Dis..

[6] Dirat B., Bochet L., Dabek M., Daviaud D., Dauvillier S., Majed B., Wang Y.Y., Meulle A., Salles B., Le Gonidec S. (2011). Cancer-associated adipocytes exhibit an activated phenotype and contribute to breast cancer invasion. Cancer Res..

[7] De Simone V., Franze E., Ronchetti G., Colantoni A., Fantini M.C., Di Fusco D., Sica G.S., Sileri P., MacDonald T.T., Pallone F. (2015). Th17-type cytokines, IL-6 and TNF-alpha synergistically activate STAT3 and NF-kB to promote colorectal cancer cell growth. Oncogene.

[8] Hodge D.R., Hurt E.M., Farrar W.L. (2005). The role of IL-6 and STAT3 in inflammation and cancer. Eur. J. Cancer.

[9] Banerjee K., Resat H. (2016). Constitutive activation of STAT3 in breast cancer cells: A review. Int. J. Cancer.

[10] Wang Y., Kuhajda F.P., Li J.N., Pizer E.S., Han W.F., Sokoll L.J., Chan D.W. (2001). Fatty acid synthase (FAS) expression in human breast cancer cell culture supernatants and in breast cancer patients. Cancer Lett..

[11] Berndt J., Kovacs P., Ruschke K., Kloting N., Fasshauer M., Schon M.R., Korner A., Stumvoll M., Bluher M. (2007). Fatty acid synthase gene expression in human adipose tissue: Association with obesity and type 2 diabetes. Diabetologia.

[12] Wang D., Dubois R.N. (2012). Associations between obesity and cancer: The role of fatty acid synthase. J. Natl. Cancer Inst..

[13] Iyengar P., Combs T.P., Shah S.J., Gouon-Evans V., Pollard J.W., Albanese C., Flanagan L., Tenniswood M.P., Guha C., Lisanti M.P. (2003). Adipocyte-secreted factors synergistically promote mammary tumorigenesis through induction of anti-apoptotic transcriptional programs and proto-oncogene stabilization. Oncogene.

[14] Kalupahana N.S., Moustaid-Moussa N. (2012). The adipose tissue renin-angiotensin system and metabolic disorders: A review of molecular mechanisms. Crit. Rev. Biochem. Mol. Biol..

[15] Jing F., Mogi M., Horiuchi M. (2013). Role of renin–angiotensin–aldosterone system in adipose tissue dysfunction. Mol. Cell. Endocrinol..

[16] Ramalingam L., Menikdiwela K., LeMieux M., Dufour J.M., Kaur G., Kalupahana N., Moustaid-Moussa N. (2017). The renin angiotensin system, oxidative stress and mitochondrial function in obesity and insulin resistance. Biochim. et Biophys. Acta Mol. Basis Dis..

[17] Namazi S., Rostami-Yalmeh J., Sahebi E., Jaberipour M., Razmkhah M., Hosseini A. (2014). The role of captopril and losartan in prevention and regression of tamoxifen-induced resistance of breast cancer cell line MCF-7: An in vitro study. Biomed. Pharmacother..

[18] Muscella A., Greco S., Elia M.G., Storelli C., Marsigliante S. (2002). Angiotensin II stimulation of Na+/K+ATPase activity and cell growth by calcium-independent pathway in MCF-7 breast cancer cells. J. Endocrinol..

[19] Pinter M., Jain R.K. (2017). Targeting the renin-angiotensin system to improve cancer treatment: Implications for immunotherapy. Sci. Transl. Med..

[20] Rodrigues-Ferreira S., Nahmias C. (2015). G-protein coupled receptors of the renin-angiotensin system: New targets against breast cancer?. Front. Pharmacol..

[21] Ni H., Rui Q., Zhu X., Yu Z., Gao R., Liu H. (2017). Antihypertensive drug use and breast cancer risk: A meta-analysis of observational studies. Oncotarget.

[22] Ulu A., Harris T.R., Morisseau C., Miyabe C., Inoue H., Schuster G., Dong H., Iosif A.M., Liu J.Y., Weiss R.H. (2013). Anti-inflammatory effects of omega-3 polyunsaturated fatty acids and soluble epoxide hydrolase inhibitors in angiotensin-II-dependent hypertension. J. Cardiovasc. Pharmacol..

[23] Fabian C.J., Kimler B.F., Hursting S.D. (2015). Omega-3 fatty acids for breast cancer prevention and survivorship. Breast Cancer Res..

[24] Kalupahana N.S., Claycombe K., Newman S.J., Stewart T., Siriwardhana N., Matthan N., Lichtenstein A.H., Moustaid-Moussa N. (2010). Eicosapentaenoic Acid Prevents and Reverses Insulin Resistance in High-Fat Diet-Induced Obese Mice via Modulation of Adipose Tissue Inflammation. J. Nutr..

[25] Kalupahana N.S., Claycombe K.J., Moustaid-Moussa N. (2011). (n-3) Fatty Acids Alleviate Adipose Tissue Inflammation and Insulin Resistance: Mechanistic Insights. Adv. Nutr..

[26] Al-Jawadi A., Moussa H., Ramalingam L., Dharmawardhane S., Gollahon L., Gunaratne P., Layeequr Rahman R., Moustaid-Moussa N. (2018). Protective properties of n-3 fatty acids and implications in obesity-associated breast cancer. J. Nutr. Biochem..

[27] Al-Jawadi A., Rasha F., Ramalingam L., Alhaj S., Moussa H., Gollahon L., Dharmawardhane S., Moustaid-Moussa N. (2020). Protective effects of eicosapentaenoic acid in adipocyte-breast cancer cell cross talk. J. Nutr. Biochem..

[28] Freund A., Jolivel V., Durand S., Kersual N., Chalbos D., Chavey C., Vignon F., Lazennec G. (2004). Mechanisms underlying differential expression of interleukin-8 in breast cancer cells. Oncogene.

[29] Siriwardhana N., Kalupahana N.S., Fletcher S., Xin W., Claycombe K.J., Quignard-Boulange A., Zhao L., Saxton A.M., Moustaid-Moussa N. (2012). n-3 and n-6 polyunsaturated fatty acids differentially regulate adipose angiotensinogen and other inflammatory adipokines in part via NF-κB-dependent mechanisms. J. Nutr. Biochem..

[30] Brinton E.A., Mason R.P. (2017). Prescription omega-3 fatty acid products containing highly purified eicosapentaenoic acid (EPA). Lipids Health Dis..

[31] Superko H.R., Superko A.R., Lundberg G.P., Margolis B., Garrett B.C., Nasir K., Agatston A.S. (2014). Omega-3 Fatty Acid Blood Levels Clinical Significance Update. Curr. Cardiovasc. Risk Rep..

[32] Itakura H., Yokoyama M., Matsuzaki M., Saito Y., Origasa H., Ishikawa Y., Oikawa S., Sasaki J., Hishida H., Kita T. (2011). Relationships between plasma fatty acid composition and coronary artery disease. J. Atheroscler. Thromb..

[33] Braeckman R.A., Stirtan W.G., Soni P.N. (2014). Pharmacokinetics of Eicosapentaenoic Acid in Plasma and Red Blood Cells After Multiple Oral Dosing With Icosapent Ethyl in Healthy Subjects. Clin. Pharmacol. Drug Dev..

[34] Song J., Li C., Lv Y., Zhang Y., Amakye W.K., Mao L. (2017). DHA increases adiponectin expression more effectively than EPA at relative low concentrations by regulating PPARγ and its phosphorylation at Ser273 in 3T3-L1 adipocytes. Nutr. Metab..

[35] Mansara P.P., Deshpande R.A., Vaidya M.M., Kaul-Ghanekar R. (2015). Differential Ratios of Omega Fatty Acids (AA/EPA+DHA) Modulate Growth, Lipid Peroxidation and Expression of Tumor Regulatory MARBPs in Breast Cancer Cell Lines MCF7 and MDA-MB-231. PLoS ONE.

[36] Cunha J.P. Consumer_Captopril_Capoten. https://www.rxlist.com/consumer_captopril_capoten/drugs-condition.htm.

[37] Small W., James J.L., Moore T.D., Fintel D.J., Lutz S.T., Movsas B., Suntharalingam M., Garces Y.I., Ivker R., Moulder J. (2018). Utility of the ACE Inhibitor Captopril in Mitigating Radiation-associated Pulmonary Toxicity in Lung Cancer: Results From NRG Oncology RTOG 0123. Am. J. Clin. Oncol..

[38] Guglin M., Munster P., Fink A., Krischer J. (2017). Lisinopril or Coreg CR in reducing cardiotoxicity in women with breast cancer receiving trastuzumab: A rationale and design of a randomized clinical trial. Am. Heart J..

[39] Onoyama K., Hirakata H., Iseki K., Fujimi S., Omae T., Kobayashi M., Kawahara Y. (1981). Blood concentration and urinary excretion of captopril (SQ 14,225) in patients with chronic renal failure. Hypertension.

[40] Iyengar N.M., Gucalp A., Dannenberg A.J., Hudis C.A. (2016). Obesity and Cancer Mechanisms: Tumor Microenvironment and Inflammation. J. Clin. Oncol..

[41] Sagaradze G., Grigorieva O., Nimiritsky P., Basalova N., Kalinina N., Akopyan Z., Efimenko A. (2019). Conditioned Medium from Human Mesenchymal Stromal Cells: Towards the Clinical Translation. Int. J. Mol. Sci..

[42] Nieman K.M., Romero I.L., Van Houten B., Lengyel E. (2013). Adipose tissue and adipocytes support tumorigenesis and metastasis. Biochim. Biophys. Acta.

[43] Carter J.C., Church F.C. (2012). Mature breast adipocytes promote breast cancer cell motility. Exp. Mol. Pathol..

[44] Vazquez-Martin A., Colomer R., Brunet J., Lupu R., Menendez J.A. (2008). Overexpression of fatty acid synthase gene activates HER1/HER2 tyrosine kinase receptors in human breast epithelial cells. Cell Prolif..

[45] Alwarawrah Y., Hughes P., Loiselle D., Carlson D.A., Darr D.B., Jordan J.L., Xiong J., Hunter L.M., Dubois L.G., Thompson J.W. (2016). Fasnall, a Selective FASN Inhibitor, Shows Potent Anti-tumor Activity in the MMTV-Neu Model of HER2(+) Breast Cancer. Cell Chem. Biol..

[46] Faggioli L., Costanzo C., Merola M., Bianchini E., Furia A., Carsana A., Palmieri M. (1996). Nuclear factor kappa B (NF-kappa B), nuclear factor interleukin-6 (NFIL-6 or C/EBP beta) and nuclear factor interleukin-6 beta (NFIL6-beta or C/EBP delta) are not sufficient to activate the endogenous interleukin-6 gene in the human breast carcinoma cell line MCF-7. Comparative analysis with MDA-MB-231 cells, an interleukin-6-expressing human breast carcinoma cell line. Eur. J. Biochem..

[47] Chavey C., Muhlbauer M., Bossard C., Freund A., Durand S., Jorgensen C., Jobin C., Lazennec G. (2008). Interleukin-8 expression is regulated by histone deacetylases through the nuclear factor-kappaB pathway in breast cancer. Mol. Pharmacol..

[48] Bravata V., Minafra L., Forte G.I., Cammarata F.P., Russo G., Di Maggio F.M., Augello G., Lio D., Gilardi M.C. (2017). Cytokine profile of breast cell lines after different radiation doses. Int. J. Radiat. Biol..

[49] Trebble T., Arden N.K., Stroud M.A., Wootton S.A., Burdge G.C., Miles E.A., Ballinger A.B., Thompson R.L., Calder P.C. (2003). Inhibition of tumour necrosis factor-a and interleukin-6 production by mononuclear cells following dietary fish-oil supplementation in healthy men and response to antioxidant co-supplementation. Br. J. Nutr..

[50] Duvall M.G., Levy B. (2016). DHA- and EPA-derived resolvins, protectins, and maresins in airway inflammation. Eur. J. Pharmacol..

[51] Illan-Cabeza N.A., Jimenez-Pulido S.B., Hueso-Urena F., Ramirez-Exposito M.J., Sanchez-Sanchez P., Martinez-Martos J.M., Moreno-Carretero M.N. (2018). Effects on estrogen-dependent and triple negative breast cancer cells growth of Ni(II), Zn(II) and Cd(II) complexes with the Schiff base derived from pyridine-2-carboxaldehyde and 5,6-diamino-1,3-dimethyluracil explored through the renin-angiotensin system (RAS)-regulating aminopeptidases. J. Inorg. Biochem..

[52] Brown I., Lee J., Sneddon A.A., Cascio M.G., Pertwee R.G., Wahle K.W., Rotondo D., Heys S.D. (2019). Anticancer effects of n-3 EPA and DHA and their endocannabinoid derivatives on breast cancer cell growth and invasion. Prostaglandins Leukot. Essent. Fat. Acids.

[53] Weng W.H., Leung W.H., Pang Y.J., Kuo L.W., Hsu H.H. (2018). EPA significantly improves anti-EGFR targeted therapy by regulating miR-378 expression in colorectal cancer. Oncol. Lett..

[54] Niazi Z.R., Silva G.C., Ribeiro T.P., Leon-Gonzalez A.J., Kassem M., Mirajkar A., Alvi A., Abbas M., Zgheel F., Schini-Kerth V.B. (2017). EPA:DHA 6:1 prevents angiotensin II-induced hypertension and endothelial dysfunction in rats: Role of NADPH oxidase- and COX-derived oxidative stress. Hypertens. Res. Off. J. Jpn. Soc. Hypertens..

[55] Ulu A., Stephen Lee K.S., Miyabe C., Yang J., Hammock B.G., Dong H., Hammock B.D. (2014). An omega-3 epoxide of docosahexaenoic acid lowers blood pressure in angiotensin-II-dependent hypertension. J. Cardiovasc. Pharmacol..

[56] Coussens L.M., Werb Z. (2002). Inflammation and cancer. Nature.

[57] Chang Q., Bournazou E., Sansone P., Berishaj M., Gao S.P., Daly L., Wels J., Theilen T., Granitto S., Zhang X. (2013). The IL-6/JAK/Stat3 Feed-Forward Loop Drives Tumorigenesis and Metastasis. Neoplasia.

[58] Krusche B., Arend J., Efferth T. (2013). Synergistic inhibition of angiogenesis by artesunate and captopril in vitro and in vivo. Evid. Based Complement. Alternat. Med..

[59] Miguel-Carrasco J.L., Zambrano S., Blanca A.J., Mate A., Vazquez C.M. (2010). Captopril reduces cardiac inflammatory markers in spontaneously hypertensive rats by inactivation of NF-kB. J. Inflamm..

[60] Lee M.J., Fried S.K. (2014). Optimal protocol for the differentiation and metabolic analysis of human adipose stromal cells. Methods Enzymol..

[61] Wortman P., Miyazaki Y., Kalupahana N.S., Kim S., Hansen-Petrik M., Saxton A.M., Claycombe K.J., Voy B.H., Whelan J., Moustaid-Moussa N. (2009). n3 and n6 polyunsaturated fatty acids differentially modulate prostaglandin E secretion but not markers of lipogenesis in adipocytes. Nutr. Metab..

