# Supplementary material for: Combined Effects of Eicosapentaenoic Acid and Adipocyte Renin–Angiotensin System Inhibition on Breast Cancer Cell Inflammation and Migration
Source: Cancers (Basel). 2020 Jan 16;12(1):220. doi: 10.3390/cancers12010220 (PMC7016836; doi:10.3390/cancers12010220)
Supplement: Supplementary file 1 [file cancers-12-00220-s001.pdf]

Supplementary tables and experimental design for EPA and CAP interactions in adipocytes and breast cancer cell crosstalk

Table 1: Exploratory factorial regression analyses to examine CAP and EPA interactions of mRNA markers associated with MDA-MB-231 cell growth and inflammation

Marker	IVs	Betas	SE	T-value	p-value
FASN	Intercept	1	0.13677	7.311526	3.84E-08
	CM	4.659069	0.205156	22.70994	1.88E-20
	EPA	-0.33801	0.193422	-1.74751	0.090779
	CAP	-0.14089	0.193422	-0.7284	0.472019
	CM:EPA	-4.73081	0.281959	-16.7784	8.61E-17
	CM:CAP	-4.41591	0.281959	-15.6615	5.54E-16
	EPA:CAP	-0.04515	0.281959	-0.16011	0.873865
	**CM:EPA:CAP**	**4.482303**	**0.398751**	**11.24087**	**2.80E-12**

Multiple R-squared: 0.969194; F-statistic: 134.835on 7 and 30 degrees of freedom

Table 2: Exploratory factorial regression analyses to examine CAP and EPA interactions of mRNA markers associated with MDA-MB-231 cell growth and inflammation

Marker	IVs	Betas	SE	T-value	p-value
STAT3	Intercept	1	0.089959	11.11617	2.41E-12
	CM	2.182367	0.134939	16.17304	1.16E-16
	EPA	-0.0585	0.127221	-0.45983	0.648844
	CAP	-0.11335	0.127221	-0.89098	0.379801
	CM:EPA	-2.13889	0.185455	-11.5332	9.57E-13
	CM:CAP	-2.0826	0.185455	-11.2296	1.87E-12
	EPA:CAP	0.162369	0.179918	0.90246	0.373771
	**CM:EPA:CAP**	**1.760098**	**0.258388**	**6.811848**	**1.25E-07**

Multiple R-squared: 0.936673; F-statistic: 65.50367 on 7 and 31 degrees of freedom

Table 3: Exploratory factorial regression analyses to examine CAP and EPA interactions of mRNA markers associated with MDA-MB-231 cell growth and inflammation

Marker	IVs	Betas	SE	T-value	p-value
NF-κB	Intercept	1	0.085336	11.7184	6.38E-13
	CM	0.77946	0.128004	6.089348	9.52E-07
	EPA	-0.24332	0.120683	-2.01618	0.052517
	CAP	-0.12927	0.120683	-1.07112	0.292383
	CM:EPA	-0.46442	0.175924	-2.63989	0.012866
	CM:CAP	-0.61338	0.175924	-3.4866	0.001485
	EPA:CAP	0.147165	0.170672	0.862269	0.395161
	CM:EPA:CAP	0.28808	0.245109	1.175314	0.248818

Multiple R-squared: 0.748958; F-statistic: 13.21216 on 7 and 31 degrees of freedom

Supplementary tables and experimental design for EPA and CAP interactions in adipocytes and breast cancer cell crosstalk

Table 4: Exploratory factorial regression analyses to examine CAP and EPA interactions of mRNA markers associated with MDA-MB-231 cell growth and inflammation					
Marker	IVs	Betas	SE	T-value	p-value
IL-6	Intercept	1	0.080806	12.37536	9.70E-14
	CM	8.653	0.127765	67.72585	3.96E-36
	EPA	-0.5338	0.119854	-4.4537	9.64E-05
	CAP	-0.03896	0.119854	-0.32504	0.747265
	CM:EPA	-8.23044	0.178871	-46.0132	8.22E-31
	CM:CAP	-8.60377	0.184265	-46.6923	5.18E-31
	EPA:CAP	0.034969	0.1695	0.206305	0.83786
	**CM:EPA:CAP**	**7.800978**	**0.252962**	**30.83854**	**2.26E-25**

Multiple R-squared: 0.995743; F-statistic: 1069.25 on 7 and 32 degrees of freedom

Table 5: Exploratory factorial regression analyses to examine CAP and EPA interactions of mRNA markers associated with MDA-MB-231 cell growth and inflammation					
Marker	IVs	Betas	SE	T-value	p-value
IL-8	Intercept	1	0.037364	26.76375	5.92E-24
	CM	0.86025	0.059078	14.56135	6.34E-16
	EPA	-0.66817	0.05542	-12.0565	1.23E-13
	CAP	-0.01818	0.05542	-0.32803	0.74496
	CM:EPA	-0.18147	0.082709	-2.19414	0.035373
	CM:CAP	-0.69187	0.082709	-8.36508	1.16E-09
	EPA:CAP	0.029101	0.078375	0.371298	0.712789
	**CM:EPA:CAP**	**0.473915**	**0.115164**	**4.115145**	**0.000242**

Multiple R-squared: 0.965257; F-statistic: 130.975 on 7 and 33 degrees of freedom

Table 6: Exploratory factorial regression analyses to examine CAP and EPA interactions of protein markers associated with MDA-MB-231 cell inflammation					
Marker	IVs	Betas	SE	T-value	p-value
IL-6	Intercept	1	0.671968	1.488166	0.162508
	CM	9.992979	1.062475	9.40538	6.92E-07
	EPA	-0.48198	0.950306	-0.50718	0.621221
	CAP	0.009325	0.950306	0.009813	0.992332
	CM:EPA	-9.8201	1.502566	-6.53556	2.79E-05
	CM:CAP	-10.2927	1.502566	-6.85011	1.77E-05
	EPA:CAP	-0.00559	1.343936	-0.00416	0.996752
	**CM:EPA:CAP**	**10.19462**	**2.124949**	**4.797581**	**0.000435**

Multiple R-squared: 0.92132; F-statistic: 20.07372 on 7 and 12 degrees of freedom

Supplementary tables and experimental design for EPA and CAP interactions in adipocytes and breast cancer cell crosstalk

Table 7: Exploratory factorial regression analyses to examine CAP and EPA interactions of protein markers associated with MDA-MB-231 cell inflammation

Marker	IVs	Betas	SE	T-value	p-value
IL-8	Intercept	1	0.611433	1.635502	0.122749
	CM	7.91525	0.864697	9.153783	1.58E-07
	EPA	-0.48738	0.864697	-0.56364	0.581328
	CAP	-0.0027	0.864697	-0.00313	0.997547
	CM:EPA	-8.17419	1.297046	-6.30216	1.42E-05
	CM:CAP	-8.43167	1.222866	-6.895	5.10E-06
	EPA:CAP	0.017865	1.222866	0.014609	0.988537
	**CM:EPA:CAP**	**8.483555**	**1.782619**	**4.75904**	**0.000253**

Multiple R-squared: 0.915312; F-statistic: 23.16021 on 7 and 15 degrees of freedom

Table 8: Exploratory factorial regression analyses to examine CAP and EPA interactions of mRNA markers associated with MCF-7 cell growth and inflammation

Marker	IVs	Betas	SE	T-value	p-value
FASN	Intercept	1	0.219363	4.558658	4.40E-05
	CM	5.041204	0.322893	15.6126	4.30E-19
	EPA	-0.10044	0.322893	-0.31107	0.757287
	CAP	-0.11523	0.310226	-0.37144	0.712179
	CM:EPA	-5.18877	0.465339	-11.1505	3.94E-14
	CM:CAP	-5.13108	0.45664	-11.2366	3.09E-14
	EPA:CAP	-0.07219	0.45664	-0.15809	0.875146
	**CM:EPA:CAP**	**5.201977**	**0.658088**	**7.904679**	**7.61E-10**

Multiple R-squared: 0.910932; F-statistic: 61.36463 on 7 and 42 degrees of freedom

Table 9: Exploratory factorial regression analyses to examine CAP and EPA interactions of mRNA markers associated with MCF-7 cell growth and inflammation

Marker	IVs	Betas	SE	T-value	p-value
STAT3	Intercept	1	0.152655	6.550723	2.79E-08
	CM	1.281499	0.230793	5.552603	1.02E-06
	EPA	-0.0918	0.222531	-0.41255	0.681667
	CAP	-0.17619	0.215887	-0.81614	0.418213
	CM:EPA	-1.07134	0.338285	-3.16697	0.002601
	CM:CAP	-1.19757	0.32639	-3.66914	0.000582
	EPA:CAP	0.029257	0.310044	0.094363	0.925191
	**CM:EPA:CAP**	**1.166133**	**0.493395**	**2.363488**	**0.021953**

Multiple R-squared: 0.532763; F-statistic: 8.307471 on 7 and 51 degrees of freedom

Supplementary tables and experimental design for EPA and CAP interactions in adipocytes and breast cancer cell crosstalk

Table 10: Exploratory factorial regression analyses to examine CAP and EPA interactions of mRNA markers associated with MCF-7 cell growth and inflammation

Marker	IVs	Betas	SE	T-value	p-value
NF-κB	Intercept	1	0.101256	9.876006	1.97E-13
	CM	2.097104	0.160099	13.09879	6.06E-18
	EPA	-0.08165	0.147604	-0.55319	0.582552
	CAP	-0.13708	0.143197	-0.95731	0.342925
	CM:EPA	-2.0176	0.229227	-8.80176	8.30E-12
	CM:CAP	-2.09184	0.226414	-9.239	1.78E-12
	EPA:CAP	0.018441	0.205651	0.08967	0.928901
	**CM:EPA:CAP**	**2.086325**	**0.322193**	**6.475388**	**3.67E-08**

Multiple R-squared: 0.848026; F-statistic: 40.6547 on 7 and 51 degrees of freedom

Table 11: Exploratory factorial regression analyses to examine CAP and EPA interactions of mRNA markers associated with MCF-7 cell growth and inflammation

Marker	IVs	Betas	SE	T-value	p-value
IL-6	Intercept	1	0.051166	19.54434	2.43E-25
	CM	-0.086	0.085617	-1.00448	0.319891
	EPA	-0.29014	0.072359	-4.00975	0.000199
	CAP	-0.36603	0.072359	-5.05848	5.83E-06
	CM:EPA	-0.1487	0.117792	-1.26236	0.212561
	CM:CAP	-0.06153	0.117792	-0.52235	0.603685
	EPA:CAP	0.194159	0.102331	1.897357	0.063449
	CM:EPA:CAP	0.144999	0.164209	0.883015	0.381371

Multiple R-squared: 0.662343; F-statistic: 14.29158 on 7 and 51 degrees of freedom

Table 12: Exploratory factorial regression analyses to examine CAP and EPA interactions of mRNA markers associated with MCF-7 cell growth and inflammation

Marker	IVs	Betas	SE	T-value	p-value
IL-8	Intercept	1	0.060337	16.57348	3.53E-22
	CM	0.972652	0.095402	10.19533	6.64E-14
	EPA	-0.33343	0.087956	-3.79089	0.000398
	CAP	-0.22486	0.08533	-2.63521	0.01111
	CM:EPA	-0.93278	0.136594	-6.82881	1.01E-08
	CM:CAP	-0.93251	0.134918	-6.91166	7.51E-09
	EPA:CAP	0.125777	0.122546	1.026367	0.309562
	**CM:EPA:CAP**	**0.897947**	**0.191992**	**4.676997**	**2.17E-05**

Multiple R-squared: 0.848884; F-statistic: 40.9269 on 7 and 51 degrees of freedom

Supplementary tables and experimental design for EPA and CAP interactions in adipocytes and breast cancer cell crosstalk

Table 13: Exploratory factorial regression analyses to examine CAP and EPA interactions of protein markers associated with MCF-7 cell inflammation

Marker	IVs	Betas	SE	T-value	p-value
IL-6	Intercept	1	0.0515	19.417	5.14E-08
	EPA	-0.10153	0.07284	-1.394	2.01E-01
	CAP	-0.11597	0.07284	-1.592	1.50E-01
	EPA:CAP	-0.10814	0.103	-1.05	3.24E-01

Multiple R-squared: 0.7254; F-statistic: 7.043 on 3 and 8 degrees of freedom

Table 14: Exploratory factorial regression analyses to examine CAP and EPA interactions of protein markers associated with MCF-7 cell inflammation

Marker	IVs	Betas	SE	T-value	p-value
IL-8	Intercept	1	0.06398	15.63	2.80E-07
	EPA	-0.14349	0.09048	-1.586	1.51E-01
	CAP	-0.16202	0.09048	-1.791	1.11E-01
	EPA:CAP	0.22748	0.12796	1.778	1.13E-01

Multiple R-squared: 0.3303; F-statistic: 1.315 on 3 and 8 degrees of freedom

Table 15: Exploratory time-series regression analyses to examine CAP and EPA interactions in MDA-MB-231 cell migration

	Estimate	Std. Error	t value	Pr(>|t|)
(Intercept)	66.74926	5.4224	12.31	< 2e-16
as.factor(Group)2	23.4281	7.66843	3.055	0.0031
as.factor(Group)3	2.30795	7.66843	0.301	0.76426
as.factor(Group)4	24.12216	7.66843	3.146	0.00237
Time	-1.65706	0.21628	-7.662	4.88E-11
as.factor(Group)2:Time	-0.26607	0.30586	-0.87	0.3871
as.factor(Group)3:Time	-0.06846	0.30586	-0.224	0.82349
as.factor(Group)4:Time	-0.18902	0.30586	-0.618	0.53844

Residual standard error: 16.38 on 76 degrees of freedom;
Multiple R-squared: 0.7993, Adjusted R-squared: 0.7808;
F-statistic: 43.24 on 7 and 76 DF, p-value: < 2.2e-16;
Group1 (reference) = Control, Group2 = EPA, Group3 = CAP, Group4 = Both

Supplementary tables and experimental design for EPA and CAP interactions in adipocytes and breast cancer cell crosstalk

Table 16: Exploratory time-series regression analyses to examine CAP and EPA interactions in MDA-MB-231 cell migration

	Estimate	Std. Error	t value	Pr(>|t|)
(Intercept)	92.25178	2.01307	45.826	< 2e-16
as.factor(Group)2	10.96192	2.84691	3.85	0.000244
as.factor(Group)3	13.42635	2.84691	4.716	1.07E-05
as.factor(Group)4	12.44705	2.84691	4.372	3.85E-05
Time	-2.13325	0.08029	-26.568	< 2e-16
as.factor(Group)2:Time	0.95701	0.11355	8.428	1.67E-12
as.factor(Group)3:Time	0.81119	0.11355	7.144	4.70E-10
as.factor(Group)4:Time	0.77482	0.11355	6.824	1.88E-09

Residual standard error: 6.079 on 76 degrees of freedom;
Multiple R-squared: 0.9599, Adjusted R-squared: 0.9562;
F-statistic: 259.9 on 7 and 76 DF, p-value: < 2.2e-16;
Group1 (reference) = Control, Group2 = EPA, Group3 = CAP, Group4 = Both

Table 17: Exploratory time-series factorial regression analyses to examine CAP and EPA interactions in MDA-MB-231 cell migration

	Estimate	std. Error	t value	Pr(>|t|)
(Intercept)	66.74926	5.4224	12.31	< 2e-16
EPA	23.4281	7.66843	3.055	0.0031
CAP	2.30795	7.66843	0.301	0.7643
Time	-1.65706	0.21628	-7.662	4.88E-11
EPA:CAP	-1.61389	10.84479	-0.149	0.8821
EPA:Time	-0.26607	0.30586	-0.87	0.3871
CAP:Time	-0.06846	0.30586	-0.224	0.8235
EPA:CAP:Time	0.14552	0.43256	0.336	0.7375

Residual standard error: 16.38 on 76 degrees of freedom;
Multiple R-squared: 0.7993, Adjusted R-squared: 0.7808;
F-statistic: 43.24 on 7 and 76 DF, p-value: < 2.2e-16

Supplementary tables and experimental design for EPA and CAP interactions in adipocytes and breast cancer cell crosstalk

Table 18: Exploratory time-series factorial regression analyses to examine CAP and EPA interactions in MDA-MB-231 cell migration				
	Estimate	std. Error	t value	Pr(>|t|)
(Intercept)	92.25178	2.01307	45.826	< 2e-16
EPA	10.96192	2.84691	3.85	0.000244
CAP	13.42635	2.84691	4.716	1.07E-05
Time	-2.13325	0.08029	-26.568	< 2e-16
EPA:CAP	-11.9412	4.02614	-2.966	0.004031
EPA:Time	0.95701	0.11355	8.428	1.67E-12
CAP:Time	0.81119	0.11355	7.144	4.70E-10
EPA:CAP:Time	-0.99338	0.16059	-6.186	2.87E-08

Residual standard error: 6.079 on 76 degrees of freedom;
Multiple R-squared: 0.9599, Adjusted R-squared: 0.9562;
F-statistic: 259.9 on 7 and 76 DF, p-value: < 2.2e-16

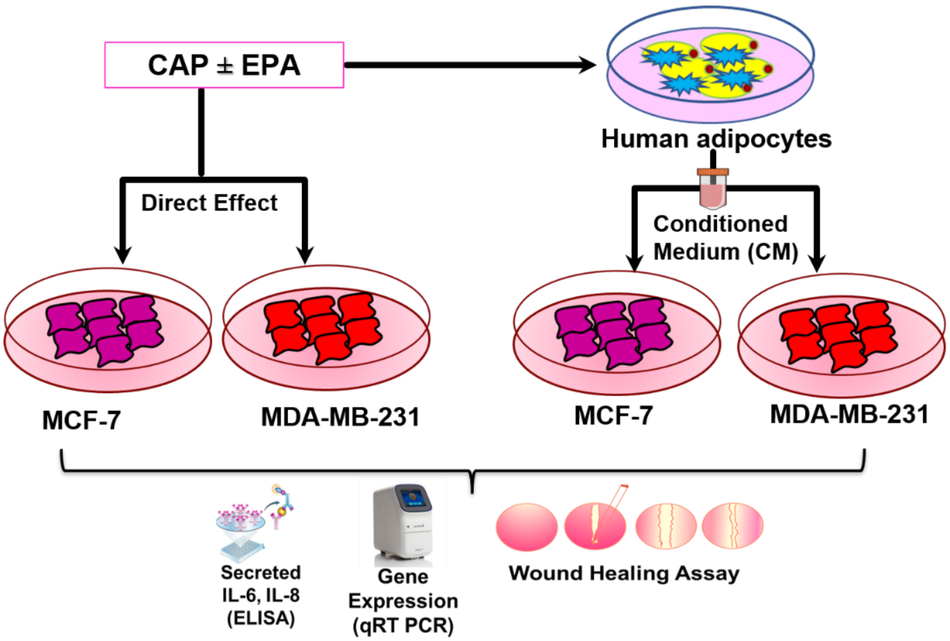

Figure S1. Experimental design. MCF-7 and MDA-MB-231 breast cancer cells were treated directly with 100 µM captopril (CAP) with and without (±) 100 µM eicosapentaenoic acid (EPA) Figure 48. hours to investigate the direct effect of CAP and EPA combinations in breast cancer. Next differentiated human adipocytes were treated with 100 µM CAP ± 100 µM EPA followed by collection conditioned medium (CM) after 24 hours. Then breast cancer cells were treated with conditioned medium to investigate the CM mediated effect of adipose RAS inhibition in breast cancer. Gene and protein analyses via qRT PCR and ELISA, and wound healing assays were performed followed by harvesting cells and medium.
